# Test–Retest Reliability and Correlates of Vertebral Bone Marrow Lipid Composition by Lipidomics Among Children With Varying Degrees of Bone Fragility

**DOI:** 10.1002/jbm4.10400

**Published:** 2020-09-08

**Authors:** Daniel G Whitney, Maureen J Devlin, Andrea I Alford, Christopher M Modlesky, Mark D Peterson, Ying Li, Michelle S Caird

**Affiliations:** ^1^ Department of Physical Medicine and Rehabilitation University of Michigan Ann Arbor MI USA; ^2^ Institute for Healthcare Policy and Innovation University of Michigan Ann Arbor MI USA; ^3^ Department of Anthropology University of Michigan Ann Arbor MI USA; ^4^ Department of Orthopaedic Surgery University of Michigan Ann Arbor MI USA; ^5^ Department of Kinesiology University of Georgia Athens GA USA

**Keywords:** BONE MARROW LIPIDS, CEREBRAL PALSY, LIPIDOMICS, RELIABILITY, SCOLIOSIS

## Abstract

The reliability of lipidomics, an approach to identify the presence and interactions of lipids, to analyze the bone marrow lipid composition among pediatric populations with bone fragility is unknown. The objective of this study was to assess the test–retest reliability, standard error of measurement (SEM), and the minimal detectable change (MDC) of vertebral bone marrow lipid composition determined by targeted lipidomics among children with varying degrees of bone fragility undergoing routine orthopedic surgery. Children aged 10 to 19 years, with a confirmed diagnosis of adolescent idiopathic scoliosis (*n* = 13) or neuromuscular scoliosis and cerebral palsy (*n* = 3), undergoing posterior spinal fusion surgery at our institution were included in this study. Transpedicular vertebral body bone marrow samples were taken from thoracic vertebrae (T11, 12) or lumbar vertebrae (L1 to L4). Lipid composition was assessed via targeted lipidomics and all samples were analyzed in the same batch. Lipid composition measures were examined as the saturated, monounsaturated, and polyunsaturated index and as individual fatty acids. Relative and absolute test–retest reliability was assessed using the intraclass correlation coefficient (ICC), SEM, and MDC. Associations between demographics and index measures were explored. The ICC, SEM, and MDC were 0.81 (95% CI, 0.55–0.93), 1.6%, and 4.3%, respectively, for the saturated index, 0.66 (95% CI, 0.25–0.87), 3.5%, and 9.7%, respectively, for the monounsaturated index, and 0.60 (95% CI, 0.17–0.84), 3.6%, and 9.9%, respectively, for the polyunsaturated index. For the individual fatty acids, the ICC showed a considerable range from 0.04 (22:2n‐6) to 0.97 (18:3n‐3). Age was positively correlated with the saturated index (*r* ^*2*^ = 0.36; *p* = 0.014) and negatively correlated with the polyunsaturated index (*r* ^*2*^ = 0.26; *p* = 0.043); there was no difference in index measures by sex (*p* > 0.58). The test–retest reliability was moderate‐to‐good for index measures and poor to excellent for individual fatty acids; this information can be used to power research studies and identify measures for clinical or research monitoring. © 2020 The Authors. *JBMR Plus* published by Wiley Periodicals LLC on behalf of American Society for Bone and Mineral Research.

## Introduction

Several investigations, some dating as far back as the 1970s, have demonstrated an inverse relationship between bone mass and bone marrow adiposity in humans and animals.^(^ ^1^, ^2^, ^3^, ^4^, ^5^, ^6^, ^7^, ^8^, ^9^ ^)^ Bone marrow pluripotent mesenchymal progenitor cells are the shared origin for osteoblasts (bone‐forming cells) and bone marrow adipocytes (lipid‐containing cells),^(^ ^10^ ^)^ among other cell lineages,^(^ ^11^ ^)^ thus giving rise to bone marrow adipose tissue at the expense of bone^(^ ^8^, ^9^, ^12^ ^)^ (and hematopoietic bone^(9^ ^)^) tissue. More recent evidence suggests that bone marrow adipose tissue possesses biological features that may be implicated in the pathogenesis of bone (and other organ) disease. As a result, there is a growing interest in bone marrow adipose tissue biology and its implications for bone and systemic energy metabolism (for reviews, please see 13, 14, 15, 16, 17, 18).^(13–18)^

The lipid composition of bone marrow is of interest, as it may serve as a key regulator in bone homeostasis. For example, some fatty acids, such as palmitic and stearic saturated fatty acids, may be lipotoxic to bone^(^ ^19^, ^20^ ^)^ and impede osteoblast function,^(^ ^21^, ^22^ ^)^ whereas other fatty acids, such as palmitoleic, oleic, and linoleic unsaturated fatty acids, may promote osteoblast differentiation, mineralization, and survival,^(^ ^20^, ^22^, ^23^ ^)^ and inhibit osteoclast differentiation and function.^(^ ^24^, ^25^ ^)^ Given the association with bone fragility,^(^ ^26^, ^27^ ^)^ the bone marrow saturated index, which is the proportion of saturated fatty acids to total fatty acids, has been suggested as a biomarker for osteoporosis.^(^ ^28^ ^)^

To date, many of the studies examining the interplay between bone and bone marrow adipose tissue have focused on adults, animal models, or in vitro experiments.^(^ ^13^, ^14^, ^15^, ^16^, ^17^, ^18^ ^)^ Growth and development is an essential window for maximizing bone health. Factors that impede typical bone acquisition during growth and development will not only diminish peak bone mass attainment, but can have lasting adverse ramifications on bone health throughout the lifespan. Accordingly, recent attention is shining the light on bone marrow adipose tissue as a potential untapped source involved in bone acquisition during development, especially for pediatric patients with varying degrees of bone fragility,^(^ ^18^, ^29^ ^)^ such as mild osteoporosis noted in the spine in adolescent idiopathic scoliosis (AIS) and the profound fragility in cerebral palsy (CP).^(^ ^30^, ^31^, ^32^ ^)^ Individuals with AIS and CP can have progressive scoliosis and may undergo spinal fusion surgery for correction of scoliosis. This provides a unique resource for acquiring pediatric bone and marrow samples to facilitate therapeutic and mechanistic research in the bone marrow adipose tissue arena with direct clinical relevance.

Lipidomics is a branch of metabolomics that seeks to identify the presence, abundance, diversity, and interactions of lipids in biological systems, and is a rapidly developing discipline making significant biomedical advances in the area of lipid biology.^(^ ^33^ ^)^ Using lipidomics to assess bone marrow lipid composition from children with AIS and CP is particularly attractive as it presents the opportunity to comprehensively analyze the bone marrow lipidome to drive research directions and address specific biological questions. To date, the reliability of lipidomics to analyze the bone marrow lipid composition among pediatric populations with bone fragility has yet to be determined. This is particularly important for bone marrow as this tissue has a highly heterogeneous cellular pool with unknown cellular and tissue‐type distribution for pediatric populations with bone fragility. Therefore, to help interpret bone marrow lipid composition measures in the clinical and research setting and to provide novel information to help power research studies where pediatric bone marrow lipid composition is a measure of interest, reliability assessment is imperative. Accordingly, the purpose of this study was to assess the test–retest reliability, SEM, and the minimal detectable change (MDC) of vertebral bone marrow lipid composition determined by targeted lipidomics among children with AIS and CP following routine orthopedic surgical care.

## Methods

### Participants

With institutional review board approval, participants aged 10 to 19 years, with a confirmed diagnosis of AIS or CP, and who were undergoing routine posterior spinal fusion surgery in the same pediatric orthopedic clinic at the University of Michigan were eligible for this study. Parental consent and, where appropriate, patient assent was obtained to use the transpedicular vertebral‐body bone‐marrow samples collected from surgery, and to obtain diagnosis and basic demographic information, including date of birth, sex, and race for all participants. Additional information was collected from children with CP, including type of CP and gross motor function classification system (GMFCS) ranking. The GMFCS ranks the severity of motor impairment on a Roman numeral scale from I to V, coinciding with mild to severe motor impairment, respectively.^(^ ^34^ ^)^ Bone marrow samples were obtained from children with spastic‐type CP and classified as GMFCS IV and V.

### Bone marrow sample extraction

Bone marrow was extracted from the vertebral body via the pedicle in the lumbar and lower thoracic spine, based on the planned spinal fusion instrumentation construct, using an 18‐gauge needle and syringe. Bone marrow aspirates were immediately placed on wet ice and kept cold until processing. Samples for lipidomics were divided into 1‐mL aliquots and stored at −80°C until analysis. Of the 34 children that met eligibility criteria for the study and had at least 1 mL of vertebral bone marrow, 16 children had two 1‐mL aliquots of bone marrow collected from the same site during the same surgery, and by the same two physicians (co‐author MSC, *n* = 15; co‐author YL, *n* = 1) from vertebrae T11 to L4 and were analyzed in this study. These two 1‐mL bone marrow aliquots are referred to as sample 1 and sample 2 hereafter. Because bone marrow tissue is heterogeneous, the research question centers on the reliability of extracting bone marrow during routine orthopedic surgery and how much variability exists in adjacent marrow tissue. Therefore, the goal of this study was to assess the reliability of different segments of the extracted marrow for lipidomics, rather than the precision or repeatability of the lipidomics technique using the same exact sample (eg, intrasample variability).

### Lipidomics

Samples were sent to the Michigan Nutrition Obesity Research Center Lipidomics Core (Ann Arbor, MI, USA) for targeted lipidomics using established procedures and the Total Lipid Extraction and Thin‐Layer (Merck, Germany) Chromatography Cleanup assay by an experienced technician. Briefly, for each bone marrow sample, lipids were extracted using a modified Bligh‐Dyer method of solvent partition.^(^ ^35^ ^)^ An aliquot of 100 μL was taken and 200 μL of water was added. Total lipids were extracted after adding 2.25 mL of chloroform‐methanol (1:2) containing 0.01% butylated hydroxytoluene and 10 μL of 4mM heptadecanoic acid (C17:0) as an internal standard. The mixtures were thoroughly homogenized on a vortex, treated with 0.75 mL of chloroform and 0.75 mL of NaCL (0.9%) solution, and then mixed and centrifuged on a table‐top centrifuge at 3,000 rpm for 15 min, after which a clear separation between the two layers was observed. The lower organic layer (chloroform) contained the lipids and was transferred into another set of tubes and saved at −20°C until further analysis.

The fatty acids were broken down into their methyl esters via transesterification with BF_3_‐methanol using a modified method, as previously described.^(^ ^36^ ^)^ The fatty acid methyl esters (FAMEs) were extracted by adding 2 mL of hexane and 1 mL of water, then mixing and centrifuging followed by collecting them in the upper hexane layers. The solvents were then removed under nitrogen and the crude FAMEs were redissolved in a small volume of chloroform and purified on a thin‐layer chromatographic plate (20 × 20 cm, silica gel 60; Merck, Darmstadt, Germany). The plate was developed with a solvent mixture of hexane‐diethyl ether‐acetic acid. Methyl ester bands were then identified by comparing the retention flow of the authentic standard and the contents from the thin‐layer chromatographic powders, and were extracted with chloroform. The solvents were removed under nitrogen and methyl esters, were redissolved in a small volume of hexane (100 to 200 μL), and the fatty acid compositions of the lipids were analyzed by gas chromatography.

Analyses of FAMEs were performed by gas chromatography on an Agilent Gas Chromatography model 6890 N (Agilent Technologies, Santa Clara, CA, USA) with a flame‐ionization detector, an auto sampler, and ChemStation software (Agilent) for analysis. The gas chromatography column used was Agilent HP 88 of 30 m, with 0.25‐mm id and 0.20‐μm film thickness. Hydrogen was used as a carrier gas as well as for the flame‐ionization detector. Nitrogen was used as a makeup gas. Analyses were carried out with a temperature program of 125°C to 220°C. One μL of sample was injected by the auto sampler, and each sample was analyzed in 20 min. A calibration curve was prepared running known amounts of methyl heptadecanoate and other commercially available standard methyl ester mixtures containing saturated and unsaturated carbon chain length from 12 through 24 carbons on gas chromatography, and using the peak area ratio response of each methyl ester with respect to methyl heptadecanoate. A mixture of authentic methyl esters was also run side‐by‐side to identify the components in unknown samples by comparing their retention times. The fatty acids were quantified with respect to the amounts of C17:0 internal standard added and the calibration curve prepared. The coefficient of variation for gas chromatography analyses was found to be within 2.3% to 3.7%.

Measures were converted to relative abundance (%) for each fatty acid and the saturated, monounsaturated, and polyunsaturated indices were calculated as the proportion of the fatty acid type (eg, saturated) to the total fatty acid amount. The nomenclature used for individual fatty acids included the number of carbon chains, the number of double bonds, and for unsaturated fatty acids, the number of carbon chains after the double‐bond and *cis* (c) or *trans* (t) configuration. For example, stearic acid 18:0, is a saturated fatty acid with 18 carbon chains and 0 double bonds; docosahexaenoic acid 22:6n‐3, is a polyunsaturated fatty acid with 22 carbon chains, six double‐bonds, and the first double‐bond at the third carbon chain from the omega end.

### Statistical analysis

Test–retest reliability was assessed using relative and absolute estimates, including the intraclass correlation coefficient (ICC; relative estimate), SEM (absolute estimate), and MDC (absolute estimate). ICC reflects the degree of correlation and agreement between measures, which is a superior method for test–retest reliability assessment.^(^ ^37^ ^)^ Following guidelines for selecting and reporting ICC for test–retest reliability,^(^ ^37^ ^)^ the ICC and 95% CI were estimated using SPSS statistical package version 24 (SPSS Inc, Chicago, IL, USA) based on a two‐way mixed‐effects model and absolute agreement. As a general rule, ICC <0.50 indicates poor reliability, ICC between 0.50 and 0.75 indicates moderate reliability, ICC between 0.75 and 0.90 indicates good reliability, and ICC >0.90 indicates excellent reliability.^(^ ^37^ ^)^ The SEM provides an estimate of the discrepancy between repeated measures or intraindividual variability,^(^ ^38^ ^)^ and was calculated as follows: SEM = SD_pooled_ × √(1‐ICC), where SD_pooled_ is the pooled SD of both sets of measures. The MDC provides an estimate of the minimal magnitude of change that is required for the measure to be considered a real change rather than the result of random variation or measurement error at the 95% CI level.^(^ ^39^, ^40^ ^)^ The MDC was calculated as follows^(^ ^41^ ^)^: MDC_95%_ = 1.96 × SEM × √2, where 1.96 comes from the *z*‐score corresponding to the 95% CI and √2 accounts for the underlying uncertainty during measurement.

### Sensitivity analysis

The reliability estimates are vulnerable to outliers and influential observations because of the small sample size. Outliers and influential observations were assessed using agreement and correlation methods for each lipid composition measure. Bland–Altman plots were constructed to evaluate the agreement between the two samples by regressing the difference between sample 1 and sample 2 measures with the mean of the two sample measures.^(^ ^42^ ^)^ The mean of the difference between bone marrow samples provides an estimate of the measure's fixed bias, which was tested statistically using a one‐sample *t* test. Normality of the mean difference was assessed using skewness, kurtosis, and the Shapiro–Wilk test.

Correlations between lipid composition measures from the two bone marrow samples were visually inspected using scatter plots. Outliers were assessed by Cook's distance (D_i_), which identifies the strength of the influence of each data point (via residuals and leverage) on the regression between measures (the higher the D_i_ value, the more influential the data point).^(^ ^43^ ^)^ Although a cutoff threshold of D_i_ > 1.0 has been proposed in the presence of a large sample size,^(^ ^44^ ^)^ we used a more conservative threshold of D_i_ > 0.50 because of the small sample size for this study. It is important to note that ICC reflects both the agreement and correlation.^(^ ^37^ ^)^ The use of Bland–Altman (agreement) and D_i_ (correlation) as model diagnostics assesses each, but not both simultaneously. Nevertheless, for lipid composition measures that displayed potential outliers or had data points with D_i_ > 0.50 (influential observations), ICC was reassessed after removing the data point(s) to determine if these values influenced the reliability estimates (assessed qualitatively).

### Exploratory analysis

We explored the association between age and sex with the index measures using bivariate regression analysis for age and the independent *t* test for sex if the data were normally distributed, or the Mann–Whitney U test if the data were nonnormally distributed. Other variables of interest (eg, CP versus AIS, race) had too few cases to perform analyses.

Data were analyzed using SPSS statistical package version 24 (SPSS Inc). An α level of 0.05 was used as the threshold to determine statistical significance.

## Results

Descriptive characteristics of the 16 participants are presented in Table 1. Notably, three of the 16 participants had spastic quadriplegic CP and were classified as GMFCS IV (*n* = 1) or V (*n* = 2), which is indicative of severe motor impairment. Of the three participants with CP, the bone marrow samples came from L2 (*n* = 1) and L4 (*n* = 2). Of the 13 participants with AIS, the bone marrow samples came from T11 (*n* = 4), T12 (*n* = 4), L1 (*n* = 3), L2 (*n* = 1), and L4 (*n* = 1).

**Table 1:** Descriptive Characteristics of Participants (*n* = 16)

Cerebral palsy, *n* (%)	3 (18.8)
GMFCS, *n*	
I–III	0
IV	1
V	2
Adolescent idiopathic scoliosis, *n* (%)	13 (81.2)
Age, mean (SD)	15.1 (1.9)
Sex, *n* (%)	
Female	9 (56.2)
Male	7 (43.8)
Race, *n* (%)	
White	13 (81.2)
Black	2 (12.5)
Refused	1 (6.3)
Bone marrow samples	
Thoracic (T)	8
T11	4
T12	4
Lumbar (L)	8
L1	3
L2	2
L3	0
L4	3

GMFCS = Gross motor function classification system.

The mean (± SD), ICC, SEM, and MDC of the lipid composition measures from the 16 bone marrow samples are presented in Table 2, which is organized based on index versus individual fatty acids, type of fatty acid (eg, saturated), and the ICC point estimate from high to low. Based on the average of the first measurement (average of second measurement), 31.1% (31.4%) of the bone marrow lipids consisted of saturated fatty acids, while 33.9% (34.6%) and 34.9% (34.0%) consisted of monounsaturated and polyunsaturated fatty acids, respectively. 18:2n‐6, 18:1n‐9, and 16:0 were the most abundant fatty acids and 13:0, 12:0, and 22:2n‐6 were the least abundant fatty acids.

**Table 2:** Relative and Absolute Test–Retest Reliability Estimates of Lipid Composition Measures From 16 Vertebral Bone Marrow Sample Pairs

	1st sample mean ± SD (%)	2nd sample mean ± SD (%)	ICC (95% CI)	SEM (%)	MDC (%)
Index measures					
Saturated index	31.07 ± 3.57	31.43 ± 3.53	0.81 (0.55, 0.93)	1.55	4.29
Monounsaturated index	33.94 ± 5.95	34.56 ± 6.09	0.66 (0.25, 0.87)	3.51	9.73
Polyunsaturated index	34.99 ± 5.73	34.01 ± 5.54	0.60 (0.17, 0.84)	3.56	9.88
Saturated fatty acids					
15:0	0.10 ± 0.09	0.10 ± 0.09	0.93 (0.82, 0.98)	0.02	0.07
18:0	9.77 ± 1.78	10.08 ± 1.79	0.76 (0.44, 0.91)	0.87	2.42
24:0	0.55 ± 0.25	0.53 ± 0.27	0.76 (0.43, 0.91)	0.13	0.35
16:0	19.39 ± 2.26	19.44 ± 2.54	0.75 (0.42, 0.91)	1.20	3.33
20:0	0.17 ± 0.07	0.19 ± 0.06	0.67 (0.29, 0.87)	0.04	0.10
14:0	0.59 ± 0.42	0.58 ± 0.37	0.64 (0.21, 0.86)	0.24	0.66
22:0	0.30 ± 0.10	0.30 ± 0.10	0.58 (0.12, 0.83)	0.06	0.18
12:0	0.01 ± 0.01	0.01 ± 0.01	0.46 (−0.04, 0.77)	0.01	0.02
19:0	0.14 ± 0.04	0.14 ± 0.03	0.42 (−0.11, 0.75)	0.03	0.07
21:0	0.05 ± 0.04	0.06 ± 0.05	0.10 (−0.42, 0.56)	0.04	0.12
13:0	<0.01 ± <0.01	<0.01 ± <0.01	NA	NA	NA
Monounsaturated fatty acids					
24:1	0.63 ± 0.40	0.61 ± 0.33	0.88 (0.69, 0.96)	0.13	0.35
14:1n‐5	0.11 ± 0.14	0.1 ± 0.09	0.87 (0.67, 0.95)	0.04	0.11
16:1n‐7 t	0.27 ± 0.06	0.26 ± 0.06	0.66 (0.26, 0.87)	0.03	0.10
18:1n‐9	24.17 ± 9.64	25.83 ± 7.93	0.57 (0.13, 0.83)	5.76	15.97
16:1n‐7c	1.65 ± 0.58	1.61 ± 0.49	0.56 (0.09, 0.82)	0.35	0.98
18:1n‐7	6.59 ± 5.71	5.54 ± 3.36	0.53 (0.07, 0.81)	3.11	8.62
22:1	0.07 ± 0.04	0.08 ± 0.03	0.35 (−0.12, 0.71)	0.03	0.08
20:1	0.46 ± 0.12	0.53 ± 0.17	0.25 (−0.21, 0.64)	0.13	0.35
Polyunsaturated fatty acids					
18:3n‐3	1.54 ± 2.26	1.39 ± 1.81	0.97 (0.92, 0.99)	0.35	0.98
22:5n‐3	0.71 ± 0.39	0.72 ± 0.53	0.85 (0.63, 0.95)	0.18	0.49
22:6n‐3	0.75 ± 0.41	0.82 ± 0.62	0.81 (0.54, 0.93)	0.22	0.62
22:4n‐6	0.62 ± 0.27	0.61 ± 0.31	0.80 (0.51, 0.93)	0.13	0.36
20:4n‐6	4.46 ± 1.67	4.32 ± 1.66	0.68 (0.29, 0.88)	0.94	2.61
20:3n‐6	0.87 ± 0.33	0.84 ± 0.30	0.67 (0.28, 0.87)	0.18	0.50
18:2n‐6	25.49 ± 3.66	24.8 ± 2.69	0.58 (0.14, 0.83)	2.06	5.70
18:3n‐6	0.14 ± 0.08	0.13 ± 0.06	0.53 (0.07, 0.81)	0.05	0.13
20:5n‐3	0.20 ± 0.33	0.15 ± 0.23	0.42 (−0.08, 0.75)	0.21	0.59
20:2	0.19 ± 0.12	0.21 ± 0.14	0.33 (−0.19, 0.70)	0.11	0.29
22:2n‐6	0.03 ± 0.02	0.03 ± 0.02	0.04 (−0.49, 0.52)	0.02	0.05

ICC = intraclass correlation coefficient; MDC = minimal detectable change; NA = not available (based on low detection).

**Fig 1:**
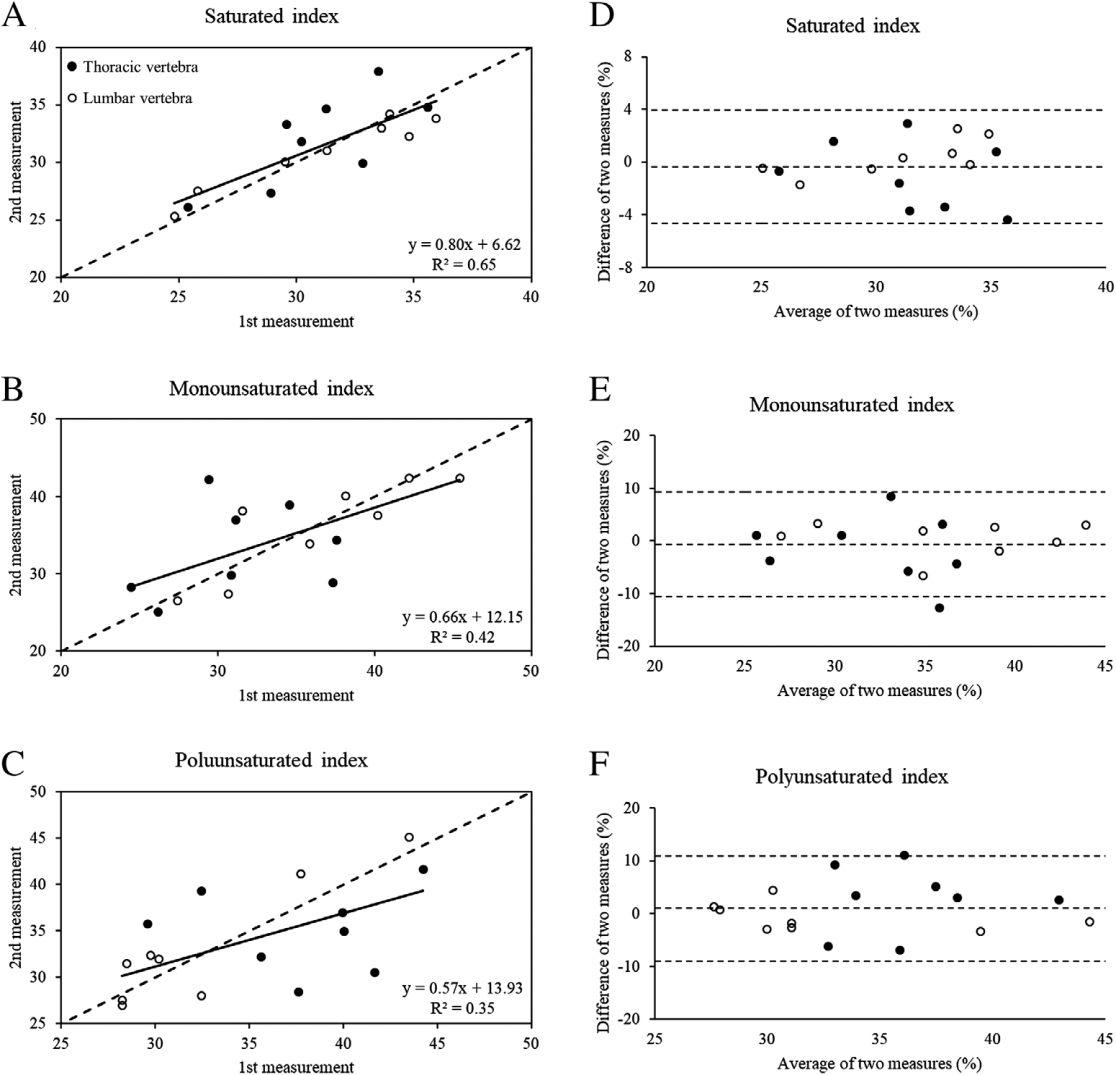
Scatter plots show the correlation (*A–C*) and Bland–Altman plots show the agreement (*D–F*) for the lipid composition index measures for thoracic (closed circles) and lumbar (open circles) marrow samples. (A–C) The dotted line represents the line of identity and the solid line represents the regression line for the combined marrow samples (ie, not stratified by thoracic or lumbar). (*D–F*) The dotted lines represent the mean difference between marrow sample pairs ±1.96 SD.

For the index measures, the ICC was 0.81 (95% CI, 0.55–0.93) for the saturated index, suggesting good (moderate‐to‐excellent) reliability, and 0.66 (95% CI, 0.25–0.87) and 0.60 (95% CI, 0.17–0.84) for the monounsaturated and polyunsaturated indices, respectively, suggesting moderate (poor‐to‐good) reliability. The SEM was 1.6%, 3.5%, and 3.6% for the saturated, monounsaturated, and polyunsaturated index, respectively, and the MDC was 4.3%, 9.7%, and 9.9%, respectively. Figure 1 shows the scatter plots demonstrating the relationship between the index measures (Fig. 1*A–C*) and Bland–Altman plots for the agreement (Fig. 1*D–F*) between bone marrow samples for the index measures. Based on the Bland–Altman plots, the average bias was minimal (near zero) for each index measure, and there was no evidence of fixed bias (all *p* > 0.45 using a one‐sample *t* test).

**Fig 2:**
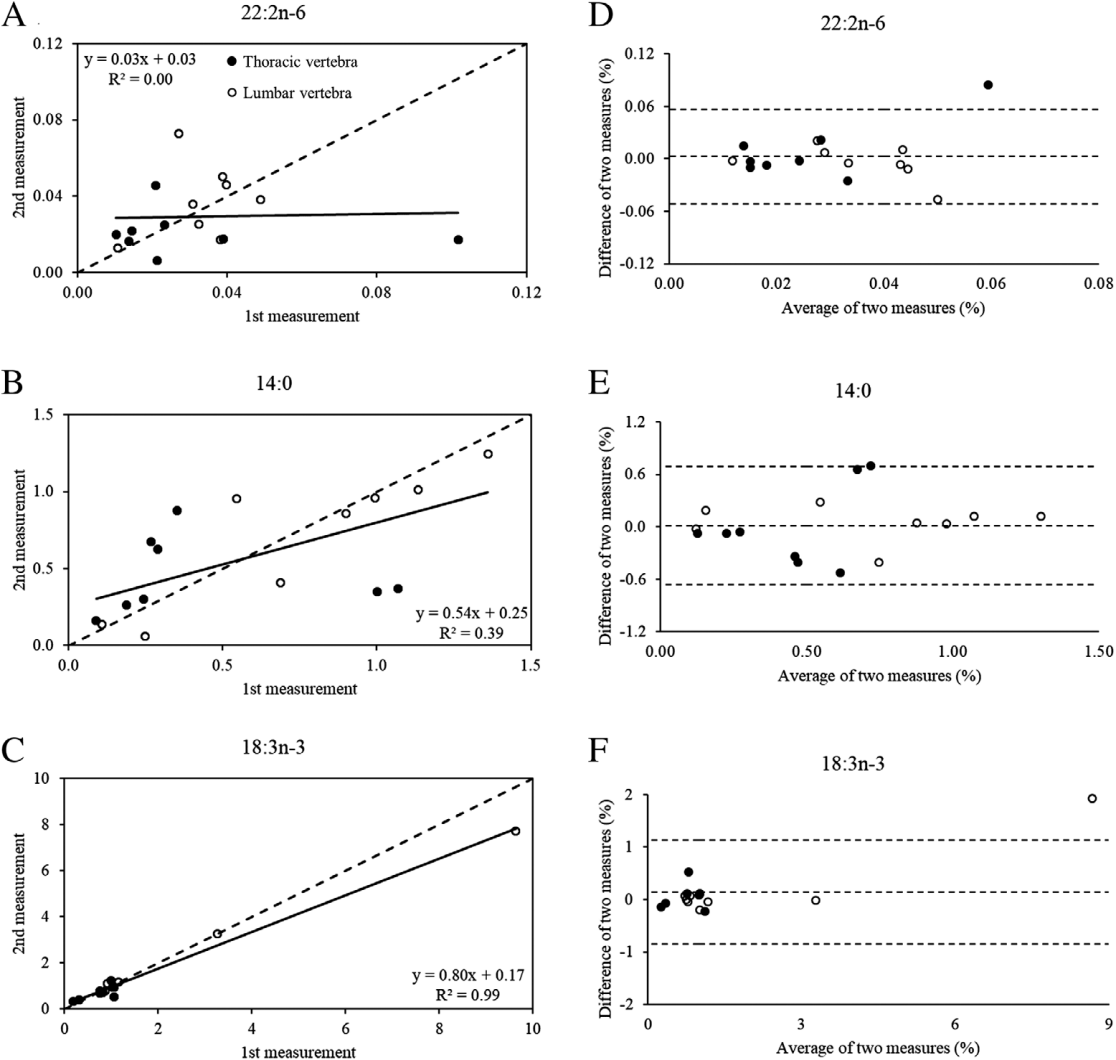
Scatter plots show the correlation (*A–C*) and Bland–Altman plots show the agreement (*D–F*) for three fatty acids representing the lowest (*A,D*), middle (*B,E*), and highest (*C,F*) reliability based on the intraclass correlation coefficient for thoracic (closed circles) and lumbar (open circles) marrow samples. (*A–C*) The dotted line represents the line of identity and the solid line represents the regression line for the combined marrow samples (ie, not stratified by thoracic or lumbar). (*D–F*) The dotted lines represent the mean difference between marrow sample pairs ±1.96 SD.

The relative abundance of 13:0 was too low to detect and assess reliability. For all other fatty acids, the ICC exhibited a considerable range from 0.04 (95% CI, −0.49 to 0.52) to 0.97 (95% CI, 0.92–0.99). Based on the ICC point estimate of the 29 fatty acids where reliability estimates were obtained (ie, excluding 13:0), 2 had excellent reliability (6.9%), 7 had good reliability (24.1%), 12 had moderate reliability (41.4%), and 8 had poor reliability (27.6%). Figure 2 shows the scatter plots (Fig. 2*A–C*) and Bland–Altman plots (Fig. 2*D–F*) of three fatty acids corresponding to the lowest (22:2n‐6), middle (14:0), and highest (18:3n‐3) ICC to represent the range of correlation and agreement among individual fatty acids. Based on the Bland–Altman plots, the average bias for these three representative fatty acids was minimal (near zero) and there was no evidence of fixed bias (all *p* > 0.27 using a one‐sample *t* test). However, the mean difference was not normally distributed for 22:2n‐6 (*p* = 0.013) or 18:3n‐3 (*p* < 0.001), and showed evidence of an outlier.

### Sensitivity analysis

In addition to 22:2n‐6 and 18:3n‐3, there was evidence of an outlier or influential observation for 12 other individual fatty acids, but not for the index measures. However, only 1 or 2 out of the 16 data points suggested a potential outlier or influential observation per fatty acid.

The mean (± SD), ICC, SEM, and MDC for the 14 fatty acids after removing the potential outliers or influential observations are presented in Table 3. The ICC value increased in six of the fatty acids, decreased in six of the fatty acids, and did not change or exhibited negligible change in two of the fatty acids. Some of the ICC values changed more drastically than others, eg, ICC increased from 0.67 (95% CI, 0.29–0.87) to 0.92 (95% CI, 0.77–0.97) for 20:0, whereas 22:5n‐3 decreased from 0.85 (95% CI, 0.63–0.95) to 0.74 (95% CI, 0.38–0.90). Based on the ICC point estimate of the 29 fatty acids where reliability estimates were obtained (ie, excluding 13:0) and incorporating the values from the sensitivity analysis, 4 had excellent reliability (13.8%; 6.9% from primary analysis), 6 had good reliability (20.7%; 24.1% from primary analysis), 11 had moderate reliability (37.9%; 41.4% from primary analysis), and 8 had poor reliability (27.6%; same as primary analysis).

**Table 3:** Relative and Absolute Test–Retest Reliability Estimates of Lipid Composition Measures From Vertebral Bone Marrow Sample Pairs After Removing Potential Outliers and Influential Observations

	1st sample mean ± SD (%)	2nd sample mean ± SD (%)	ICC (95% CI)	SEM (%)	MDC (%)
Saturated fatty acids					
20:0 (*n* = 15)	0.18 ± 0.06	0.19 ± 0.06	0.92 (0.77, 0.97)	0.02	0.05
19:0 (*n* = 15)	0.14 ± 0.04	0.15 ± 0.03	0.64 (0.21, 0.86)	0.02	0.06
21:0 (*n* = 15)	0.05 ± 0.04	0.05 ± 0.02	0.57 (0.09, 0.83)	0.02	0.05
12:0 (*n* = 14)	<0.01 ± <0.01	<0.01 ± 0.01	0.10 (−0.43, 0.58)	<0.01	0.01
Monounsaturated fatty acids					
18:1n‐9 (*n* = 15)	25.48 ± 8.38	25.27 ± 7.88	0.91 (0.75, 0.97)	2.44	6.76
14:1n‐5 (*n* = 15)	0.08 ± 0.08	0.08 ± 0.07	0.85 (0.60, 0.95)	0.03	0.08
16:1n‐7 t (*n* = 15)	0.26 ± 0.04	0.25 ± 0.05	0.35 (−0.21, 0.73)	0.04	0.10
18:1n‐7 (*n* = 14)	4.52 ± 0.71	4.72 ± 0.48	0.17 (−0.38, 0.63)	0.54	1.50
Polyunsaturated fatty acids					
18:3n‐3 (*n* = 15)	1.00 ± 0.68	0.97 ± 0.69	0.97 (0.91, 0.99)	0.12	0.33
20:5n‐3 (*n* = 14)	0.09 ± 0.11	0.11 ± 0.17	0.88 (0.66, 0.96)	0.05	0.13
22:5n‐3 (*n* = 15)	0.64 ± 0.28	0.62 ± 0.34	0.74 (0.38, 0.90)	0.16	0.44
22:4n‐6 (*n* = 15)	0.58 ± 0.23	0.55 ± 0.24	0.71 (0.33, 0.89)	0.13	0.35
22:2n‐6 (*n* = 15)	0.03 ± 0.01	0.03 ± 0.02	0.35 (−0.19, 0.73)	0.01	0.03
18:3n‐6 (*n* = 15)	0.13 ± 0.07	0.12 ± 0.04	0.34 (−0.19, 0.72)	0.04	0.12

ICC = intraclass correlation coefficient; MDC = minimal detectable change.

### Exploratory analysis

**Fig 3:**
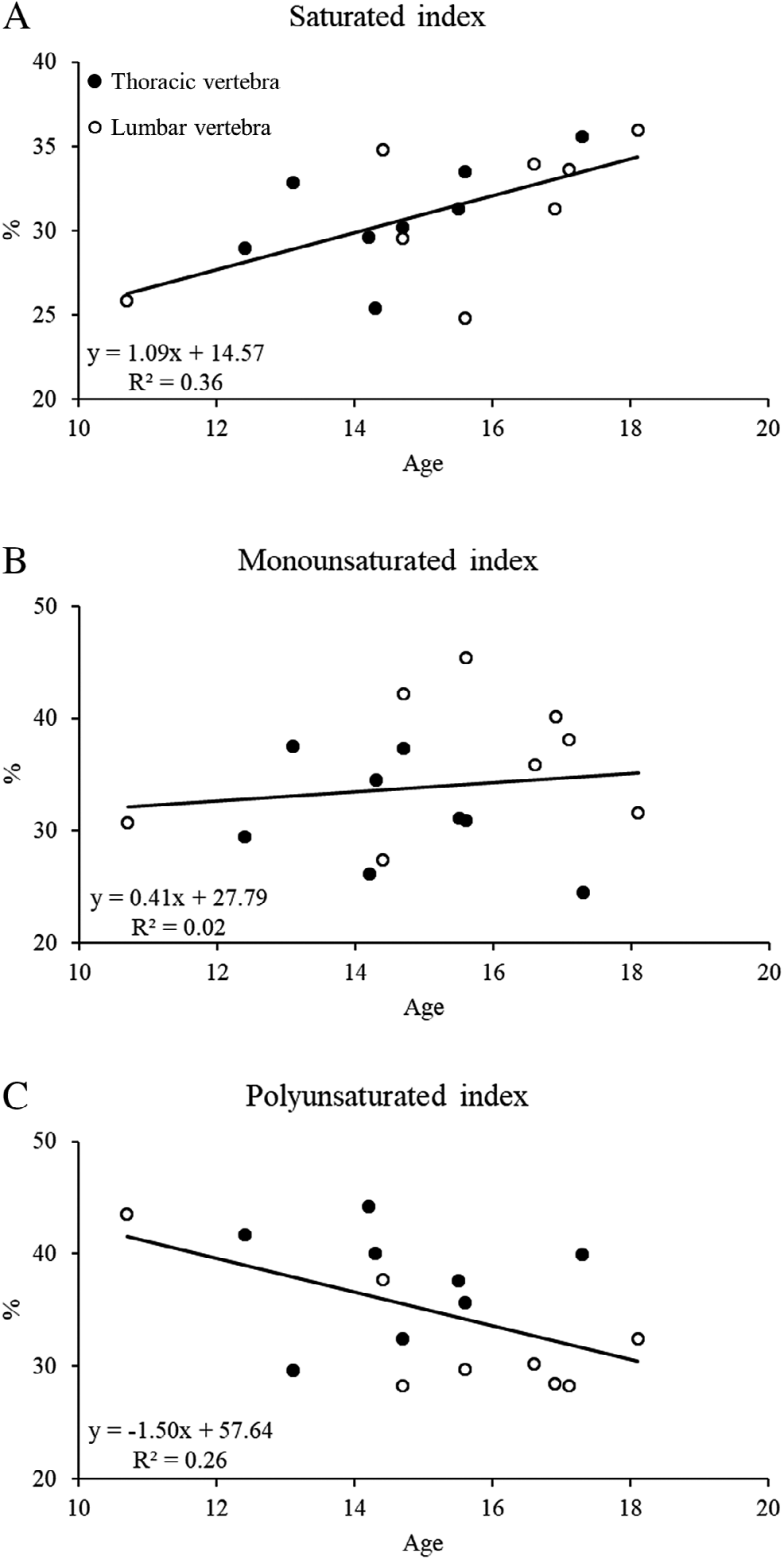
Scatter plots show the correlation between age with the saturated index (*A*), monounsaturated index (*B*), and polyunsaturated index (*C*) for thoracic (closed circles) and lumbar (open circles) marrow samples. The solid line represents the regression line for the combined marrow samples (ie, not stratified by thoracic or lumbar).

Age was significantly and positively correlated with the saturated index (Fig. 3A, *r* ^*2*^ = 0.36; *p* = 0.014), not significant correlated with the monounsaturated index (Fig. 3B), and significantly and negatively correlated with the polyunsaturated index (Fig. 3C, *r* ^*2*^ = 0.26, *p* = 0.043; results are presented for sample 1, but are consistent for both samples). There was no statistical difference between girls and boys for the saturated index (30.84 ± 2.86 versus 31.37 ± 4.55; *p* = 0.78), monounsaturated index (34.68 ± 5.00 versus 32.98 ± 7.31; *p* = 0.59), polyunsaturated index (34.48 ± 6.15 versus 35.65 ± 5.54; *p* = 0.70), or age (14.74 ± 1.52 versus 15.50 ± 2.46; *p* = 0.46) for this small sample (index results are presented for sample 1, but are consistent for both samples); however, all nine girls had AIS whereas three out of seven of the boys had CP, and bone marrow was mostly extracted from the thoracic spine for girls (*n* = 6) and for boys it was the lumbar spine (*n* = 5).

## Discussion

The findings from this study suggest moderate‐to‐good test–retest reliability for the vertebral bone marrow saturated and unsaturated index via targeted lipidomics from a small sample of children with AIS and CP using bone marrow collected under routine orthopedic surgical conditions. When we examined individual fatty acids, we observed a considerable range from poor‐to‐excellent test–retest reliability with approximately one third having good to excellent (34.5%), moderate (37.9%), or poor (27.6%) test–retest reliability. In the exploratory analysis, we found that age was positively correlated with the saturated index and negatively correlated with the polyunsaturated index. These findings have important logistical implications for future research and clinical endeavors because delineating the bone marrow lipid composition from pediatric populations with varying degrees of bone fragility may provide further insight into the role of bone marrow adipose tissue on bone and systemic energy metabolism. This may be missed by only investigating the extent of fatty infiltration, although if and how bone marrow lipids contribute to skeletal acquisition and metabolism among children and adolescents is unknown.

In the current study, we observed that the test–retest reliability of some fatty acids were sensitive to potential outliers or influential observations. Specifically, in the sensitivity analysis, we observed one or two potential outliers or influential observations for four saturated fatty acids, four monounsaturated fatty acids, and six polyunsaturated fatty acids exhibiting a range in relative abundance (<0.01% to 25.48%). It is important to note that these potential outliers or influential observations came from 10 different participants rather than the same or few participants, which may suggest the potential for considerable biological variability in bone marrow lipids between and within participants. Although bone marrow consists of multiple cellular lineages, the cellular and tissue heterogeneity may be even greater or unique for individuals with bone fragility, such as AIS and CP. Factors related to AIS and CP that may influence the status of bone marrow lipid composition, and its variability within a given site, include nutrition, hormonal factors, comorbidities (eg, cardiometabolic disease, inflammation), surgeries, and medications.^(^ ^18^, ^45^, ^46^ ^)^ However, it is important to note that the variability may be attributed solely to or in conjunction with the small sample size; we therefore urge caution in interpretation. Further, the current study found that age during this adolescent period was associated with a higher bone marrow saturated index and a lower polyunsaturated index, but none of these indices was associated with sex. These findings are consistent with studies that found higher marrow fat with age in the distal femur of 11‐ to 18‐year‐olds with anorexia nervosa^(^ ^47^ ^)^ and in L4 of typically developing newborns to 18‐year‐olds with no evidence of sex differences.^(^ ^48^ ^)^

The reasons for the suboptimal reliability performance for some individual fatty acids are likely multifactorial. The relative abundance may be one of these reasons. Although not presented in the Results section, when the relationship between the relative abundance of sample 1 and the ICC value was examined for all 30 individual fatty acids, the *r* ^*2*^ was 0.01 (*p* = 0.530). However, when only fatty acids with less than 1% abundance were included (*n* = 22), there was a stronger and positive correlation with the *r* ^*2*^ at 0.32 (*p* = 0.006). This may be because of logistical considerations for the fatty acids with very low abundance, such as detection sensitivity of the lipidomics technique or sample collection or processing methods that may elicit degradation of these fatty acids. For example, bone marrow fatty acid integrity may be impacted by collection methods (eg, needle extraction may damage cell membranes), storage temperature (eg, colder environments may limit enzyme activity and lipid degradation), duration of storage, and the number of freeze–thaw cycles,^(^ ^49^, ^50^, ^51^, ^52^, ^53^, ^54^, ^55^ ^)^ especially for fatty acids with very low abundance. None of the samples in the current study underwent previous freeze–thaw cycles. Nevertheless, study findings have important implications for research and clinical investigation where biological effects of specific fatty acids from bone marrow may be of interest. Based on our findings using bone marrow collected during routine orthopedic surgical conditions, which may not be generalizable to other clinical populations or methods that are designed specifically for bone marrow extraction for analysis, we urge caution when assessing specific fatty acids with very low abundance from bone marrow based on the potential for poor reliability in measurement assessment.

We observed that the SEM was 1.6%, 3.5%, and 3.6% for the saturated, monounsaturated, and polyunsaturated index, respectively, and that the SEM ranged from <0.1% to 5.8% for the individual fatty acids. These observations indicate the range of values around the point estimate for each measure for intraindividual variability.^(^ ^38^ ^)^ We observed that the MDC was 4.3%, 9.7%, and 9.9% for the saturated, monounsaturated, and polyunsaturated index, respectively, and that the MDC ranged from <0.1% to 16.0% for the individual fatty acids. These findings suggest that a change between repeated measurements larger than the MDC values could indicate a real change with 95% certainty among a similar sample. Seeing the considerable range of MDC, the degree of change needed to be considered a true change depends on the specific bone marrow lipid‐composition measure. These values can be used for power analysis and sample‐size calculation for research studies. Further consideration for longitudinal assessment is that the absolute abundance of some individual fatty acids may remain the same, whereas others change drastically, thus impacting the relative abundance measure. How the interplay between relative and absolute measures impacts biological outcomes remains to be determined.

It is important to note that there are other approaches to examine the composition of bone marrow that are less invasive. For example, bone marrow fat content using magnetic resonance spectroscopy has been used as a biomarker to distinguish skeletal fragility and altered metabolic states, such as type 2 diabetes mellitus,^(^ ^27^, ^56^ ^)^ and standard MRI has been used to quantify the extent of bone marrow fat infiltration in pediatric populations.^(^ ^30^ ^)^ A major advantage of in vivo imaging is, in general, the relatively high reproducibility, such as an ICC of 0.96 for bone marrow fat content in children with and without CP.^(^ ^30^ ^)^ However, the in vivo imaging techniques that are currently available for human research lack more granular information that tissue extraction can provide, such as the degree of saturation and unsaturation using magnetic resonance spectroscopy^(^ ^27^, ^47^, ^56^ ^)^ compared with quantifying individual fatty acids using tissue lipidomics.

A major strength of this study is that, to our knowledge, this is the first study to assess the bone marrow lipid composition in children with varying degrees of bone fragility, and the first study to assess the test–retest reliability of bone marrow lipid profiles among children and adolescents. Further, the interest in the bone marrow adipose field is growing, yet the acquisition of bone marrow tissue from children and adolescents for research is rare. Therefore, this work provides novel and fundamental insights about the interpretability of bone marrow lipidomics among pediatric populations with bone fragility, and the use of human tissue facilitates data acquisition with direct clinical relevance. Although meaningful patterns did emerge, one limitation of this study is the small sample size. However, a large sample size is not feasible because of the invasive nature of bone marrow acquisition. Nevertheless, the findings from this sample size have practical implications as research studies that include clinical pediatric populations, such as AIS or CP, often have few participants caused by logistical challenges, such as a low recruitment pool. Therefore, a sample size of 16 is not uncommon for such studies, especially for studies that require tissue extraction. Another limitation is the unknown site‐specific effect on reliability measures as we collected tissue from T11 to L4. However, even if there are differences in bone marrow lipid profiles along the vertebral column, the potential variability is less for reliability assessment in this study considering the tissue samples are collected from the same site (as opposed to intersite), immediately adjacent to one another, and collected during the same surgery. In this study, site‐specific variation of reliability would indicate vertebral differences in the degree of bone marrow tissue heterogeneity. Although it is well‐documented that bone marrow tissue is highly heterogeneous in terms of a diverse cellular pool, we are unaware of any studies that have shown that the degree of heterogeneity differs by neighboring vertebral sites. Further, we examined the correlation and agreement by lumbar and thoracic sites (eg, all figures), which provides little evidence of site‐specific effects. However, the sample size is small; therefore, we are unable to conclusively determine the presence or absence of site‐specific effects on the reliability measures, SEM, and MDC. Finally, our exploratory analyses examined a small set of potential variables, which may be confounded by other age‐related factors, such as height.

In conclusion, the test–retest reliability for vertebral bone marrow saturated and unsaturated index was moderate‐to‐good and poor‐to‐excellent for individual fatty acids, using targeted lipidomics from children with bone fragility in which bone marrow was collected during routine orthopedic surgery. Further, age, but not sex, was associated with a higher and lower saturated and polyunsaturated index, respectively, but the lack of sex difference may have been caused by differences in characteristics (eg, CP). Future studies are needed to determine the interplay among bone, marrow, physiology, physical function, and disease for pediatric populations with bone fragility, not just for reliability assessment of the bone marrow lipidome, but also for clinical management, intervention, and optimizing peak bone mass attainment for these skeletally vulnerable populations.

## Disclosures

All authors state that they have no conflicts of interest.

## Author Contributions

**Daniel Whitney:** Data curation; funding acquisition; resources; methodology; data analysis; writing‐ initial draft and editing. **Maureen Devlin:** Data curation; funding acquisition; resources; writing‐review and editing. **Andrea Alford:** Funding acquisition; writing‐review and editing. **Christopher Modlesky:** Methodology; writing‐review and editing. **Mark Peterson:** Funding acquisition; writing‐review and editing. **Ying Li:** Data curation; resources; writing‐review and editing. **Michelle Caird:** Data curation; funding acquisition; resources; writing‐review and editing.

### Peer Review

The peer review history for this article is available at https://publons.com/publon/10.1002/jbm4.10400.

## References

[1] Meunier P , Aaron J , Edouard C , Vignon G . Osteoporosis and the replacement of cell populations of the marrow by adipose tissue. A quantitative study of 84 iliac bone biopsies. Clin Orthop Relat Res. 1971;80:147–54. 5133320 10.1097/00003086-197110000-00021

[2] Minaire P , Edouard C , Arlot M , Meunier PJ . Marrow changes in paraplegic patients. Calcif Tissue Int. 1984;36(3):338–40. 6432298 10.1007/BF02405340

[3] Martin RB , Zissimos SL . Relationships between marrow fat and bone turnover in ovariectomized and intact rats. Bone. 1991;12(2):123–31. 2064840 10.1016/8756-3282(91)90011-7

[4] Di Iorgi N , Mo AO , Grimm K , Wren TA , Dorey F , Gilsanz V . Bone acquisition in healthy young females is reciprocally related to marrow adiposity. J Clin Endocrinol Metab. 2010;95(6):2977–82. 20392872 10.1210/jc.2009-2336 PMC2902071

[5] Di Iorgi N , Rosol M , Mittelman SD , Gilsanz V . Reciprocal relation between marrow adiposity and the amount of bone in the axial and appendicular skeleton of young adults. J Clin Endocrinol Metab. 2008;93(6):2281–6. 18381577 10.1210/jc.2007-2691 PMC2435643

[6] Wren TA , Chung SA , Dorey FJ , Bluml S , Adams GB , Gilsanz V . Bone marrow fat is inversely related to cortical bone in young and old subjects. J Clin Endocrinol Metab. 2011;96(3):782–6. 21177790 10.1210/jc.2010-1922

[7] Shen W , Velasquez G , Chen J , et al. Comparison of the relationship between bone marrow adipose tissue and volumetric bone mineral density in children and adults. J Clin Densitom. 2014;17(1):163–9. 23522982 10.1016/j.jocd.2013.02.009 PMC3770790

[8] Cohen A , Dempster DW , Stein EM , et al. Increased marrow adiposity in premenopausal women with idiopathic osteoporosis. J Clin Endocrinol Metab. 2012;97(8):2782–91. 22701013 10.1210/jc.2012-1477 PMC3410269

[9] Justesen J , Stenderup K , Ebbesen EN , Mosekilde L , Steiniche T , Kassem M . Adipocyte tissue volume in bone marrow is increased with aging and in patients with osteoporosis. Biogerontology. 2001;2(3):165–71. 11708718 10.1023/a:1011513223894

[10] David V , Martin A , Lafage‐Proust MH , et al. Mechanical loading down‐regulates peroxisome proliferator‐activated receptor gamma in bone marrow stromal cells and favors osteoblastogenesis at the expense of adipogenesis. Endocrinology. 2007;148(5):2553–62. 17317771 10.1210/en.2006-1704

[11] Chamberlain G , Fox J , Ashton B , Middleton J . Concise review: mesenchymal stem cells: their phenotype, differentiation capacity, immunological features, and potential for homing. Stem Cells. 2007;25(11):2739–49. 17656645 10.1634/stemcells.2007-0197

[12] Syed FA , Oursler MJ , Hefferanm TE , Peterson JM , Riggs BL , Khosla S . Effects of estrogen therapy on bone marrow adipocytes in postmenopausal osteoporotic women. Osteoporos Int. 2008;19(9):1323–30. 18274695 10.1007/s00198-008-0574-6 PMC2652842

[13] Lecka‐Czernik B . Marrow fat metabolism is linked to the systemic energy metabolism. Bone. 2012;50(2):534–9. 21757043 10.1016/j.bone.2011.06.032 PMC3197966

[14] Fazeli PK , Horowitz MC , MacDougald OA , et al. Marrow fat and bone—new perspectives. J Clin Endocrinol Metab. 2013;98(3):935–45. 23393168 10.1210/jc.2012-3634 PMC3590487

[15] Devlin MJ , Rosen CJ . The bone‐fat interface: basic and clinical implications of marrow adiposity. Lancet Diabetes Endocrinol. 2015;3(2):141–7. 24731667 10.1016/S2213-8587(14)70007-5 PMC4138282

[16] During A , Penel G , Hardouin P . Understanding the local actions of lipids in bone physiology. Prog Lipid Res. 2015;59:126–46. 26118851 10.1016/j.plipres.2015.06.002

[17] Scheller EL , Cawthorn WP , Burr AA , Horowitz MC , MacDougald OA . Marrow adipose tissue: trimming the fat. Trends Endocrinol Metab. 2016;27(6):392–403. 27094502 10.1016/j.tem.2016.03.016 PMC4875855

[18] Whitney DG , Peterson MD , Devlin MJ , Caird MS , Hurvitz EA , Modlesky CM . Bone marrow fat physiology in relation to skeletal metabolism and cardiometabolic disease risk in children with cerebral palsy. Am J Phys Med Rehabil. 2018;97(12):911–9. 29894311 10.1097/PHM.0000000000000981 PMC6237626

[19] Alsahli A , Kiefhaber K , Gold T , et al. Palmitic acid reduces circulating bone formation markers in obese animals and impairs osteoblast activity via C16‐ceramide accumulation. Calcif Tissue Int. 2016;98(5):511–9. 26758875 10.1007/s00223-015-0097-z

[20] Gillet C , Spruyt D , Rigutto S , et al. Oleate abrogates palmitate‐induced lipotoxicity and proinflammatory response in human bone marrow‐derived mesenchymal stem cells and osteoblastic cells. Endocrinology. 2015;156(11):4081–93. 26327577 10.1210/en.2015-1303

[21] Gunaratnam K , Vidal C , Gimble JM , Duque G . Mechanisms of palmitate‐induced lipotoxicity in human osteoblasts. Endocrinology. 2014;155(1):108–16. 24169557 10.1210/en.2013-1712

[22] Elbaz A , Wu X , Rivas D , Gimble JM , Duque G . Inhibition of fatty acid biosynthesis prevents adipocyte lipotoxicity on human osteoblasts in vitro. J Cell Mol Med. 2010;14(4):982–91. 19382912 10.1111/j.1582-4934.2009.00751.x PMC2891630

[23] Platt ID , El‐Sohemy A . Regulation of osteoblast and adipocyte differentiation from human mesenchymal stem cells by conjugated linoleic acid. J Nutr Biochem. 2009;20(12):956–64. 19019668 10.1016/j.jnutbio.2008.08.008

[24] Rahman MM , Halade GV , Williams PJ , Fernandes G . t10c12‐CLA maintains higher bone mineral density during aging by modulating osteoclastogenesis and bone marrow adiposity. J Cell Physiol. 2011;226(9):2406–14. 21660964 10.1002/jcp.22578 PMC3103755

[25] van Heerden B , Kasonga A , Kruger MC , Coetzee M . Palmitoleic acid inhibits RANKL‐induced osteoclastogenesis and bone resorption by suppressing NF‐kappaB and MAPK signalling pathways. Nutrients. 2017;9(5):1–14. 10.3390/nu9050441 PMC5452171 28452958

[26] Yeung DK , Griffith JF , Antonio GE , Lee FK , Woo J , Leung PC . Osteoporosis is associated with increased marrow fat content and decreased marrow fat unsaturation: a proton MR spectroscopy study. J Magn Reson Imaging. 2005;22(2):279–85. 16028245 10.1002/jmri.20367

[27] Patsch JM , Li X , Baum T , et al. Bone marrow fat composition as a novel imaging biomarker in postmenopausal women with prevalent fragility fractures. J Bone Miner Res. 2013;28(8):1721–8. 23558967 10.1002/jbmr.1950 PMC3720702

[28] Di Pietro G , Capuani S , Manenti G , et al. Bone marrow lipid profiles from peripheral skeleton as potential biomarkers for osteoporosis: a 1H‐MR spectroscopy study. Acad Radiol. 2016;23(3):273–83. 26774740 10.1016/j.acra.2015.11.009

[29] Shiran SI , Shabtai L , Ben‐Sira L , Ovadia D , Wientroub S . T1‐weighted MR imaging of bone marrow pattern in children with adolescent idiopathic scoliosis: a preliminary study. J Child Orthop. 2018;12(2):181–6. 29707058 10.1302/1863-2548.12.180035 PMC5902753

[30] Whitney DG , Singh H , Miller F , et al. Cortical bone deficit and fat infiltration of bone marrow and skeletal muscle in ambulatory children with mild spastic cerebral palsy. Bone. 2017;94:90–7. 27732905 10.1016/j.bone.2016.10.005 PMC5912954

[31] Modlesky CM , Whitney DG , Singh H , Barbe MF , Kirby JT , Miller F . Underdevelopment of trabecular bone microarchitecture in the distal femur of nonambulatory children with cerebral palsy becomes more pronounced with distance from the growth plate. Osteoporos Int. 2015;26(2):505–12. 25199575 10.1007/s00198-014-2873-4

[32] Ishida K , Aota Y , Mitsugi N , et al. Relationship between bone density and bone metabolism in adolescent idiopathic scoliosis. Scoliosis. 2015;10:19. 26075016 10.1186/s13013-015-0043-x PMC4464881

[33] Lam SM , Shui G . Lipidomics as a principal tool for advancing biomedical research. J Genet Genomics. 2013;40(8):375–90. 23969247 10.1016/j.jgg.2013.06.007

[34] Wood E , Rosenbaum P . The gross motor function classification system for cerebral palsy: a study of reliability and stability over time. Dev Med Child Neurol. 2000;42(5):292–6. 10855648 10.1017/s0012162200000529

[35] Bligh EG , Dyer WJ . A rapid method of total lipid extraction and purification. Can J Biochem Physiol. 1959;37(8):911–7. 13671378 10.1139/o59-099

[36] Morrison WR , Smith LM . Preparation of fatty acid methyl esters + dimethylacetals from lipids with boron fluoride‐methanol. J Lipid Res. 1964;5(4):600. 14221106

[37] Koo TK , Li MY . A guideline of selecting and reporting Intraclass correlation coefficients for reliability research. J Chiropr Med. 2016;15(2):155–63. 27330520 10.1016/j.jcm.2016.02.012 PMC4913118

[38] Bueno‐Gracia E , Malo‐Urries M , Ruiz‐de‐Escudero‐Zapico A , et al. Reliability of measurement of the carpal tunnel and median nerve in asymptomatic subjects with ultrasound. Musculoskelet Sci Pract. 2017;32:17–22. 28800435 10.1016/j.msksp.2017.08.001

[39] Beninato M , Portney LG . Applying concepts of responsiveness to patient management in neurologic physical therapy. J Neurol Phys Ther. 2011;35(2):75–81. 21934362 10.1097/NPT.0b013e318219308c

[40] Fritz SL , Blanton S , Uswatte G , Taub E , Wolf SL . Minimal detectable change scores for the Wolf Motor function test. Neurorehabil Neural Repair. 2009;23(7):662–7. 19498013 10.1177/1545968309335975

[41] Haley SM , Fragala‐Pinkham MA . Interpreting change scores of tests and measures used in physical therapy. Phys Ther. 2006;86(5):735–43. 16649896

[42] Bland JM , Altman DG . Statistical methods for assessing agreement between two methods of clinical measurement. Lancet. 1986;1(8476):307–10. 2868172

[43] Cook RD . Influential observations in linear regression. J Am Stat Assoc. 1979;74(365):169–74.

[44] Cook DR . Residuals and Influence in Regression. New York, NY: Chapman & Hall; 1982.

[45] Naveiras O , Nardi V , Wenzel PL , Hauschka PV , Fahey F , Daley GQ . Bone‐marrow adipocytes as negative regulators of the haematopoietic microenvironment. Nature. 2009;460(7252):259–63. 19516257 10.1038/nature08099 PMC2831539

[46] Menagh PJ , Turner RT , Jump DB , et al. Growth hormone regulates the balance between bone formation and bone marrow adiposity. J Bone Miner Res. 2010;25(4):757–68. 19821771 10.1359/jbmr.091015 PMC3153330

[47] Ecklund K , Vajapeyam S , Mulkern RV , et al. Bone marrow fat content in 70 adolescent girls with anorexia nervosa: magnetic resonance imaging and magnetic resonance spectroscopy assessment. Pediatr Radiol. 2017;47(8):952–62. 28432403 10.1007/s00247-017-3856-3 PMC5650065

[48] Ruschke S , Pokorney A , Baum T , et al. Measurement of vertebral bone marrow proton density fat fraction in children using quantitative water‐fat MRI. MAGMA. 2017;30(5):449–60. 28382554 10.1007/s10334-017-0617-0

[49] Bitman J , Wood DL , Mehta NR , Hamosh P , Hamosh M . Lipolysis of triglycerides of human milk during storage at low temperatures: a note of caution. J Pediatr Gastroenterol Nutr. 1983;2(3):521–4. 6620059 10.1097/00005176-198302030-00021

[50] Ishikawa M , Maekawa K , Saito K , et al. Plasma and serum lipidomics of healthy white adults shows characteristic profiles by subjects' gender and age. PLoS One. 2014;9(3):e91806. 24632803 10.1371/journal.pone.0091806 PMC3954792

[51] Breier M , Wahl S , Prehn C , et al. Targeted metabolomics identifies reliable and stable metabolites in human serum and plasma samples. PLoS One. 2014;9(2):e89728. 24586991 10.1371/journal.pone.0089728 PMC3933650

[52] Yang W , Chen Y , Xi C , et al. Liquid chromatography‐tandem mass spectrometry‐based plasma metabonomics delineate the effect of metabolites' stability on reliability of potential biomarkers. Anal Chem. 2013;85(5):2606–10. 23387999 10.1021/ac303576b

[53] Taylor LA , Arends J , Hodina AK , Unger C , Massing U . Plasma lyso‐phosphatidylcholine concentration is decreased in cancer patients with weight loss and activated inflammatory status. Lipids Health Dis. 2007;6:17. 17623088 10.1186/1476-511X-6-17 PMC1939842

[54] Kudo I , Murakami M . Phospholipase A2 enzymes. Prostaglandins Other Lipid Mediat. 2002;68–69:3–58. 10.1016/s0090-6980(02)00020-5 12432908

[55] Lessig J , Fuchs B . Plasmalogens in biological systems: their role in oxidative processes in biological membranes, their contribution to pathological processes and aging and plasmalogen analysis. Curr Med Chem. 2009;16(16):2021–41. 19519379 10.2174/092986709788682164

[56] Baum T , Yap SP , Karampinos DC , et al. Does vertebral bone marrow fat content correlate with abdominal adipose tissue, lumbar spine bone mineral density, and blood biomarkers in women with type 2 diabetes mellitus? J Magn Reson Imaging. 2012;35(1):117–24. 22190287 10.1002/jmri.22757 PMC3245661

